# Advancements and challenges in robotic surgery: A holistic examination of operational dynamics and future directions

**DOI:** 10.1016/j.sipas.2025.100294

**Published:** 2025-07-06

**Authors:** Abdullah Riad, Majed Hadid, Adel Elomri, Ahmad Al-Ansari, Mohamed Amine Rejeb, Marwa Qaraqe, Sarada Parsad Dakua, Abdel Rahman Jaber, Abdulla Al-Ansari, Omar M. Aboumarzouk, Abdelfatteh EL Omri

**Affiliations:** a Division of Engineering Management and Decision Sciences, College of Science and Engineering, Hamad Bin Khalifa University, Qatar Foundation, Doha, Qatar; b Weill Cornell Medicine-Qatar, Qatar Foundation - Education City, P.O. Box 24144, Doha, Qatar; c Surgical Research Section, Department of Surgery, Hamad Medical Corporation, Doha 3050, Qatar; d Division of Information and Computing Technology, College of Science and Engineering, Hamad Bin Khalifa University, Qatar Foundation, Doha, Qatar

**Keywords:** Operations management, Optimization, Performance enhancement, Robotic surgeries, Skill assessment

## Abstract

**Background:**

In recent years, Robot-Assisted Surgeries (RAS) have advanced significantly, revolutionizing healthcare with better patient outcomes, faster recovery times, and greater surgical accuracy. However, challenges such as restricted maneuverability and communication issues persist, along with the need to evaluate surgeons' proficiency in RAS. Healthcare 5.0 seeks to enhance RAS by integrating technologies like advanced imaging, haptic feedback, and artificial intelligence.

**Objective:**

This paper explores the Operational Management of Robotic-Assisted Surgeries (OM-RAS) by analyzing recent research. It assesses surgical robotics operations, workflow optimization, performance enhancement, and skill assessment. Additionally, it examines challenges and gaps in the RAS domain, providing a comprehensive research overview.

**Methods:**

A comprehensive literature search was conducted across databases such as Scopus, Web of Science, and Google Scholar, covering publications up to the third quarter of 2023. Search terms were selected using scientific criteria and validated by experts, resulting in a substantial document collection. A rigorous screening process, aligned with PRISMA 2020 standards, filtered the selection to 50 research papers, forming the foundation for detailed investigation.

**Findings:**

OM-RAS is a multidimensional field influenced by emerging technologies that optimize workflows, enhance performance, and improve skill assessment in RAS. Despite superior outcomes, challenges such as implementation costs and seamless technology integration persist. Additionally, research gaps exist regarding RAS benefits, creating opportunities for efficiency enhancement in patient care. Further exploration of RAS procedure scheduling across different surgery types is essential.

**Conclusion:**

Integrating advanced technologies into RAS has improved surgical outcomes, shortened hospital stays, and enhanced working environments. This study advocates for a holistic multi-criteria decision-making approach, considering factors like kinematics, imaging, Internet of Things/Tactile Internet (IoT/TI), and AI. It provides valuable insights, guiding future research and shaping OM-RAS studies.

## Highlights

• This review examines operational challenges in robotic-assisted surgeries (RAS).
• Key RAS research areas include workflow, performance, and skill assessment.
• Challenges include high costs and seamless integration of new technologies.
• Research gaps exist in RAS benefits and procedure scheduling across surgeries.
• A structured decision-making approach is essential for advancing RAS.

## Graphical abstract

**Figure:**
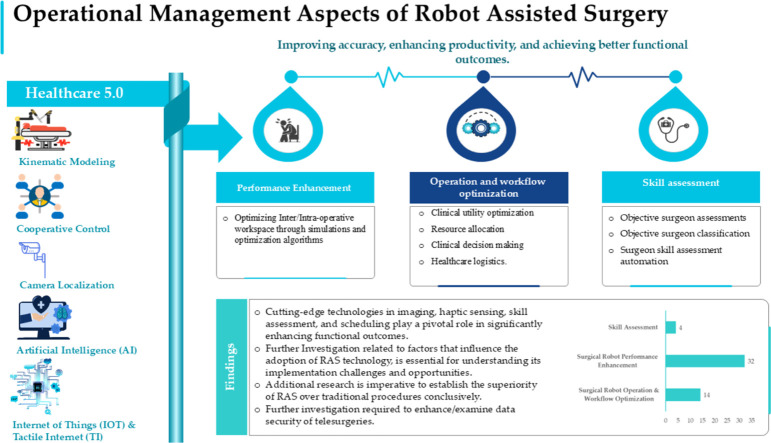

## 1 Introduction

Rapid advances in medical innovation over the last few decades have increased the number of options for disease diagnosis and treatment [1]. Consequently, innovations such as minimally invasive robotic surgeries have gained widespread recognition and popularity in recent times due to their numerous advantages. In this context, robotic surgery or Robot-Assisted Surgeries (RAS) display better functional outcomes in terms of Length of Stay (LOS) and Operative Time (OT), in addition to faster recovery, improved patient outcomes, and less post-operative pain [2].

RAS can surpass traditional surgeries in terms of performance metrics such as procedural error, economy of motion, diagnostic error, and task completion time [3]. It can also outperform both open and laparoscopic surgeries in terms of hand tremor and visual feedback, respectively. In addition to its ability to collect visual and spatial data to manage robotic system limitations and improve the surgeons’ experience [4]. It can be either teleoperated, comanipulated, supervisory-controlled, or shared-controlled. Most of the research papers focus on comanipulated or telesurgical operations since they are the most commonly performed types of robotic surgery [5].

However, RAS comes with certain drawbacks, including limitations in maneuverability, operational workspace (both inter- and intra-operative), communication among the surgical team, and assessment of surgeons’ skills in using the robot’ [[6], [7], [8]]. Also, if the surgery is teleoperated over a large distance between the surgeons and patients, some constraints such as time delay and real-time data delivery must be considered [9]. Surgical robots such as Da Vinci are not fully autonomous, so studying RAS pertains to the study of human-machine interaction. Moreover, although robotic surgeries provide more dexterity compared to traditional surgeries, they still require new technologies and innovations to further increase dexterity related to the ability to perform surgical procedures with precision.

Therefore, examining the Operational Management (OM) of robotic surgeries involves the latest technology/innovation associated with Healthcare 5.0, including advanced imaging, haptic sensing (force feedback), improved robotic articulation, sensors, and more. This examination also encompasses skill assessment as well as healthcare operations and workflow, such as resource allocation and cost of implementation. All the aforementioned collectively impact the performance of OM.

Prior reviews have explored various aspects of surgical robotics. Fruggiero et al [7] evaluated factors influencing Da Vinci robotic laparoscopic surgery, ranking them through an analytic hierarchy process and categorizing their risks. Dlaka et al [10] reviewed RAS applications in stereotactic and spinal neurosurgery for preplanning, navigation, and localization. Chioson, Espiritu, Munsayac, Jimenez, et al [11] highlighted the latest developments in surgical robots in the Philippines, discussing different systems, the current state of RAS, and recent technological advancements.

As for haptic feedback, in a comprehensive review done by M et al. [12], the authors discussed haptic gloves, which are one of the latest RAS-related technologies used to mimic the sense of touch and enhance human-machine interaction, in addition to the latest technologies related to haptic gloves. The study highlighted the methodology used in previous haptic studies and some correction measures. An overview of Artificial Intelligence (AI) in RAS has been discussed by Eminaga & Liao [4], where subjects such as RAS pre-preparation, navigation, and automated maneuverability of repetitive tasks have been highlighted. Notably, the latest generation of robotic systems from Intuitive, the Da Vinci 5, features advanced Force Feedback technology. This innovation enables surgeons to perceive push and pull forces, detect tissue tension, and experience a realistic sense of pressure during critical tasks such as dissection, retraction, and suturing, enhancing precision and control in surgical procedures.

In this context, this literature review paper is taking a unique path by examining operational management aspects of RAS (OM-RAS) through simulation and optimization. Simulations/optimization-related studies can be seen in different aspects such as resource allocation and scheduling, haptic/force sensing simulation, tele-operations of RAS, imaging, and skills assessment. Factors affecting operational management, such as time-related factors, accuracy, reliability, and other performance metrics, will be examined.

This study stands out for its innovative approach, examining both the direct and indirect factors that impact the OM-RAS. In Section 3.1: Surgical Robotics Operations and Workflow Optimization, it precisely examines the direct factors affecting OM-RAS, while 3.2, 3.3: Surgical Robotics Performance Enhancement and Skill Assessment, discuss indirect influences. Previous reviews often emphasized advancements in RAS technology, such as sensor integration for optimal positioning or algorithmic enhancements for manipulator control, alongside Convolutional Neural Networks (CNNs) for image classification improvement. However, from an operational management standpoint, these technological aspects are regarded as indirect contributors to functional outcomes of RAS, a critical aspect that this literature review aims to address.

## 2 Methodology

The methodology of this literature review paper adopted a comprehensive search strategy for the retrieval of pertinent articles from electronic databases. These databases included Scopus, Web of Science, and Google Scholar. The search was not limited to a specific time period, allowing for a comprehensive exploration across different timeframes. The selection of search terms was guided by a scientific approach, as elucidated by Chabowski et al. [13] and Zupic & Čater [14]. Moreover, the analysis incorporated insights from a thorough examination of pertinent literature review papers outlined in Table 1, with a particular emphasis on the research conducted by Moglia et al. [15]. To ensure the rigor of this search strategy, a panel of experts within the research field was engaged. Their involvement encompassed the validation of the compiled search query, the establishment of filtering criteria, the identification of supplementary search terms, and the incorporation of relevant terminology.

**Table 1:** Previous review papers.

Reference	Focus	Timespan	Size
Fruggiero et al. [7]	Variables affecting RAS	1987–2015	40
Dlaka et al. [10]	Stereotactic and spinal neurosurgery	1988–2021	38
Fuertes-Guiró et al. [16]	Opportunity cost of implementing DaVinci-RAS	1992–2013	36
M et al. [12]	Haptic glove	2004–2020	23
Moglia et al. [15]	A systematic review on artificial intelligence in robot-assisted surgery	1994–2021	78
Lam et al. [8]	Machine learning for technical skill assessment in surgery: a systematic review	1988–2021	105
Giansanti [17]	Current Trends and Future Possibilities of Integrating AI into Public Health (RAS addressed)	2021–2022	28
Moawad et al. [18]	How AI/AR affects the future of RAS and the latest technologies applied to different medical specialties	2006–2020	17
Current review	Operations Management of RAS	2006–2023	50

After iterative refinement, the search query is documented in Table 2 and the criteria for inclusion and exclusion are outlined in Table 3 were ultimately employed. The methodology adhered to the Preferred Reporting Items for Systematic Reviews and Meta-Analyses (PRISMA) 2020 statement and checklist [19]The reference 'Preferred Reporting Items for Systematic Reviews and Meta-Analyses (PRISMA) 2020′ is cited in the text but is not listed in the references list. Please either delete the in-text citation or provide full reference details following journal style., as well as the guidelines for assessing the methodological quality of systematic reviews, A Measurement Tool to Assess Systematic Reviews (AMSTAR 2) [20].

**Table 2:** Search query.

((artificial AND intelligence) OR (deep AND learning) OR (machine AND learning) OR (convolution AND neural AND network) OR (skills AND assessment) OR (plan*) OR (schedul*) OR (simulat*) OR (optimiz*) OR (optimis*) OR (operate*) OR (manage*) OR (model*) OR (program*) OR (appointment)) AND ((robotic AND surg*) OR (surgical AND robot*) OR (robot-assisted AND surg*) OR (da AND vinci AND surg*))

**Table 3:** Filtering criteria.

1. Query keywords must exist in the publication title, abstract, or keywords.2. Only retain publications that satisfy the following conditions: ○ Type: Journal article or conference paper ○ Language: English ○ Time: up to the third quarter of 2023 ○ Subject areas: “Decision Science”, “Business, Management and Accounting”, and “Economics, Econometrics and Finance”

**Fig. 1:**
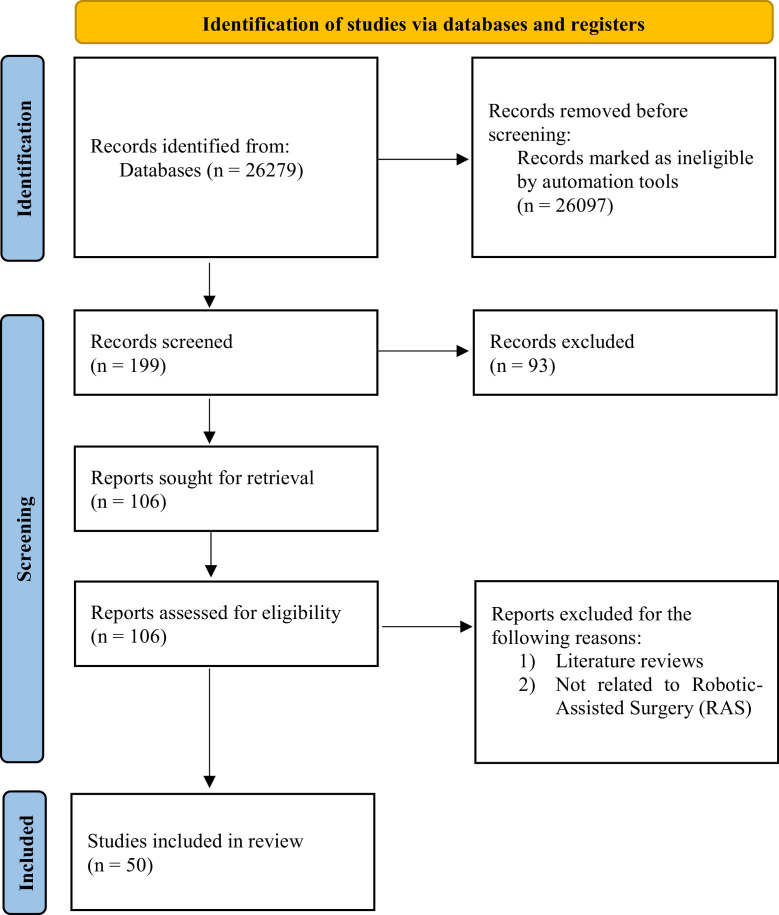
Flow Chart of the Study Selection Process Based on PRISMA.

Following the elimination of duplicate articles, a total of 178,038 papers were identified, as illustrated in Fig. 1. In order to focus exclusively on studies directly aligned with the research objectives, the search was restricted to articles that incorporated the query keywords within their titles, abstracts, and keywords, resulting in a refined dataset of 26,279 documents.

Further refinement was achieved by applying filters after reviewing articles from various research fields, such as medicine, engineering, computer science, and others. From this, specific subject areas were delineated as shown in Table 3. This step notably reduced the dataset to 199 documents. Furthermore, the search filters were used to exclusively extract conference papers and articles, excluding books, press articles, and the like, resulting in a final dataset of 190 documents. From this dataset, we identified 9 previous review papers, which we have analyzed and summarized in Table 1.

These 190 studies underwent meticulous scrutiny in Excel to facilitate manual screening. The screening process involved assessments based on titles and abstracts, complemented by comprehensive reviews of full texts when necessary. Disagreements between reviewers during the screening process were addressed through discussion, and if consensus could not be reached, a third reviewer was consulted to make the final decision. Notably, studies unrelated to the research domain, such as those related to agriculture, communication, and drilling systems, were excluded during this phase, resulting in the formation of an initial set of included studies. Subsequently, papers related to RAS were included if they demonstrated the potential to impact the operational management of RAS. Eligible studies addressed aspects such as workflow optimization, enhancement of robotic system performance, or improvement of surgeon–robot interaction, all of which are believed to contribute to better surgical processes and improved medical outcomes. To ensure maximum coverage, both backward and forward snowballing approaches were employed as suggested by Wohlin [21], leading to the identification of a few additional publications that were not initially included.

**Fig. 2:**
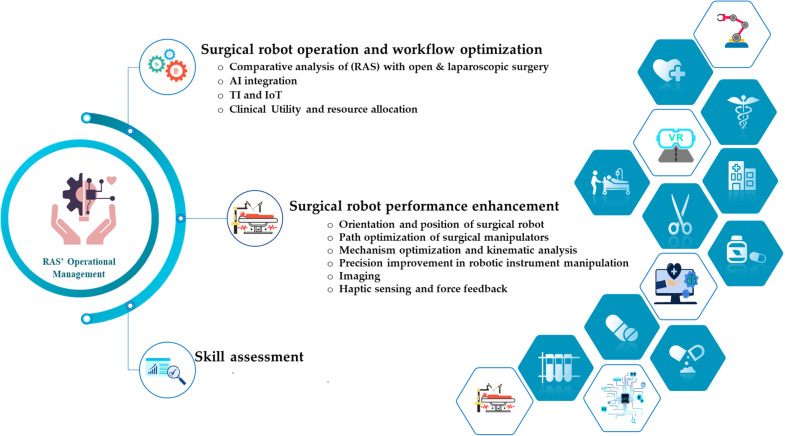
Research Aspects and Emphasis in Operational Management of Robotic-Assisted Surgery (OM-RAS).

**Fig. 3:**
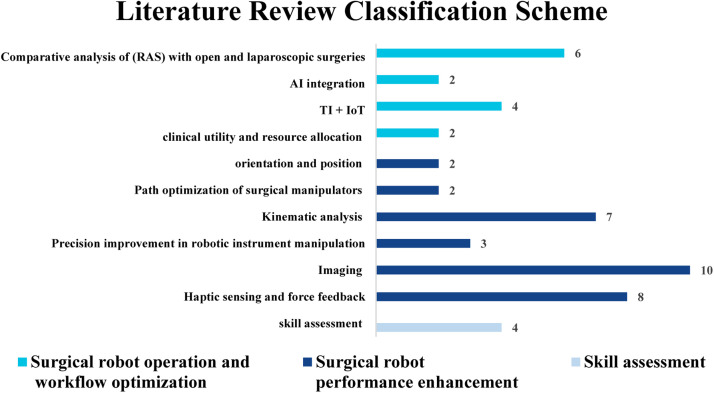
Classification Scheme of Robot-Assisted Surgery (RAS) Operations Management (OM) Literature.

Subsequently, the final dataset included 50 studies. This was followed by the thorough processes of data extraction and content analysis. The included studies have been categorized into three types: observational studies, real-world validated studies, and mixed-design studies (which combine both observational and real-world validation components). The supplementary materials of this paper include Table S1, which classifies each study according to these categories. The culmination of these analytical steps facilitated the creation of the classification schemes presented in Fig. 2, Fig. 3.

## 3 Results and discussions

The healthcare landscape is evolving rapidly, with terms like Hospitals of the Future (HoF) and Healthcare 5.0 gaining prominence. These terms underscore the growing significance of integrating the latest technological trends into our healthcare systems, as they are believed to have a profound impact on various healthcare facets, including clinical, financial, organizational, and technological outcomes [[22], [23], [24]].

As a result, the integration of RAS into our healthcare systems has emerged as a prominent trend. It is increasingly recognized that RAS can lead to improvements in peri‑operative outcomes, such as reduced patient waiting times, shorter LOS, optimized OT utilization, and minimized surgical complications (e.g., reduced Blood Loss (BL), Urinary Incontinence (UI), Urinary Complications (UC), erectile dysfunction, etc.)—as evidenced by studies [[25], [26], [27]]

However, the extent of these improvements and the specific tools and technologies employed remain key considerations. The true impact of RAS on healthcare outcomes will become clearer as we thoroughly explore the OM aspects of RAS and examine the factors influencing its performance. Consequently, this literature review paper aims to spotlight recent research that explores techniques, tools, and technologies that have the potential to influence RAS and its peri‑operative outcomes, ultimately enhancing the precision of surgical procedures.

In our examination of this section, we have summarized the OM of RAS (OM-RAS), as depicted in Fig. 2. Our findings reveal that the majority of studies within this field predominantly emphasize the latest technologies and associated simulations. Subsequently, research attention shifts towards RAS workflow optimization and performance enhancement. Skill assessment and studies related to cost-benefit analysis appear to be relatively less prevalent. This distribution highlights the evolving focus of OM-RAS research, showing a clear shift toward technological innovation and process improvement. As mentioned in the preceding section, 50 studies have been incorporated into this paper. Among these studies, 14 studies are dedicated to the optimization of surgical robotics operations and workflow (Table 5), 14 focus on the surgical robotics performance enhancement (Table 6), an additional 10 probes deeply into imaging (Table 7), while 8 address haptic sensing and force feedback (Table 8). Moreover, 4 studies center around skill assessment (Table 9). A quick summary for each subsection can be seen in Table 4.

**Table 4:** Results and discussion subsection summary.

**Subsection**	**Summary**
3.1 Surgical Robotics Operations and Workflow Optimization	It explores the improvement of various operational and medical performance metrics, including resource utilization, LOS, and OT. This subsection emphasizes the significance of integrating RAS into healthcare and clinical operations, such as the allocation of beds and operating rooms.
3.2 Surgical Robotics Performance Enhancement	Focuses on studies dedicated to enhancing the performance of surgical robotics. It includes detailed sub-sections for imaging and force/haptic feedback, as these areas have seen more research compared to other aspects of performance improvement. It also underscores the critical importance of optimizing RAS tools both intra-operatively (within the patient's body) and inter-operatively (within the operating workspace). Additionally, it reviews the significance of optimizing end-effector track and RAS mechanism/kinematics.
3.3 Skill Assessment	Focuses on simulation and optimization models related to skill assessment and haptic feedback, capitalizing on emerging technologies and Machine Learning (ML) techniques to enhance accuracy and optimize performance metrics. It is mainly about how to assess surgeon dexterity through AI algorithms.

### 3.1 Surgical robotics operations and workflow optimization

This section comprises a total of 14 studies. The first part addresses six comparative analyses of RAS with traditional procedures [[28], [29], [30], [31], [32], [33]]. Two studies that address strategic considerations concerning the implementation of RAS and AI integration [34,22]. Subsequently, the third part explores four studies digging into tactical aspects, focusing on the integration of Tactile Internet (TI) and Internet of Things (IoT) within the domain of Robot-Assisted Tele-Surgeries (RATS) workflow optimization [23,24,35,36]. Finally, the fourth section examines two studies centered on operational decisions, specifically exploring clinical utility and resource allocation [37,26].

#### 3.1.1 Comparative analysis of robot-assisted surgeries (RAS) with open and laparoscopic surgeries

Lipsitz et al. [32], using data from the Nationwide Inpatient Sample (NIS), found increased discharge hazard rates for RAS compared to open and laparoscopic approaches across nephrectomy, prostatectomy, and partial nephrectomy. Similarly, Faria et al. [30] evaluated the long-term cost-effectiveness of open, laparoscopic, and robot-assisted radical prostatectomy over 20 years using Quality-Adjusted Life-Year (QALYs), Incremental Cost-Effectiveness Ratio (ICER), and Incremental Cost-Utility Ratio (ICUR). Their analysis, which included health outcomes such as biochemical recurrence, metastasis, and post-operative complications, indicated RAS had a 32.9 % and 38.9 % chance of dominance over open and laparoscopic surgeries, respectively. However, the study’s reliance on diverse sources for intra- and peri‑operative data may limit its applicability to the Brazilian context, increasing uncertainty [30].

Al-Thani et al. [28] found robotic adrenalectomy to be safe and effective for small benign tumors (<6 cm), though surgical choice depended on factors such as tumor characteristics, patient health, surgeon expertise, and institutional resources. Open surgery remained preferred for larger or malignant tumors. The study noted potential bias due to evolving hospital practices and called for further research on costs, risks, and quality of life. In pancreatic surgery, Duran et al. [29] reported that robotic distal pancreatectomy led to significantly less blood loss and shorter hospital stays compared to open surgery, with outcomes comparable to laparoscopic procedures and a lower morbidity rate. Similarly, H.-I. Kim et al. [31] evaluated 434 gastrectomies and found comparable complication and recovery rates between robotic and laparoscopic groups. However, robotic surgeries were longer and more expensive, without perioperative advantages. In liver surgery, Yu et al. [33] found no significant differences between robotic and laparoscopic resections regarding operative time, blood loss, complications, or hospital stay.

Nevertheless, despite its advancements, robotic surgery has its limitations, including high costs and the absence of tactile feedback; such a topic is addressed in 3.2.6. Also, comparative studies between robotic and laparoscopic liver surgeries have shown comparable outcomes. However, more in-depth research encompassing diverse cases and longer follow-ups is necessary for a thorough and precise comparison. Additionally, while the observational studies offer important preliminary insights, they highlight the need for further real-world validation and long-term evaluation to strengthen the evidence base.

#### 3.1.2 Artificial intelligence (AI) integration

Combining artificial intelligence with virtual and augmented reality (AR) will enable easy access to virtual healthcare services and enhance the effectiveness and safety of robotic surgeries, which is believed to be the future trend [17].

In robotic surgery, AR improves real-time surgical performance by modifying actual environments rather than creating simulations. Integrated with telemanipulation systems, AR enhances surgeons’ visual perspectives by overlaying key anatomical landmarks during procedures, thus improving safety and peri‑operative outcomes [18]. It also helps compensate for the lack of tactile feedback by visually enriching the operative field. Additionally, AR supports clinical decision-making by displaying contextual data, such as tissue characteristics and adjacent structures [38,39].

Numerous survey studies exemplify the proof-of-concept for integrating AR into various specific RAS procedures. Several investigations showcase the feasibility of incorporating AR into a range of surgical interventions, such as partial nephrectomy [40], cholecystectomy [41], TECAB [42], and radical prostatectomy [39]. Interestingly, no complications were detected in the previous studies.

AR has its limitations. Improper registration of AR may result in displaying the tumor at an incorrect location, a phenomenon known as "misregistration". If surgeons base their surgical decisions solely on the AR display, they could unintentionally harm healthy tissue while leaving the tumor unaffected. Falk et al. [42], recorded an overlay accuracy ranging from 9.3 mm to 19.2 mm during a TECAB cardiac procedure due to heart deformation. As a result, the integration of emerging AI technologies with AR holds the promise of accurately identifying various body tissues. This can be achieved through AI algorithms leveraging deep learning-based CNNs or segmentation algorithms. Additionally, tracking algorithms can be employed to dynamically track tissues within the patient's body in real-time, enhancing the precision and effectiveness of surgical interventions and medical procedures.

Agarwal et al. [34] conducted a qualitative study at Apollo Hospital in India, emphasizing the strategic value of AI and RAS, particularly when performed by skilled surgeons using minimally invasive techniques. Through a partnership with PMI/Microsoft in 2018, the hospital leveraged large datasets to accurately predict cardiovascular risks, highlighting AI’s potential in broader applications, including reducing infection risks through RAS. The study concluded that RAS improves productivity, reduces post-operative complications, and supports faster recovery, making it a cost-effective option. Similarly, Ali Mohamad et al. [22], based on nine interviews at a Dubai hospital, found that integrating AI into robotic surgery positively influences clinical, financial, organizational, and technological outcomes.

Implementing AI encounters some challenges, notably in data collection and bias mitigation. Unbiased, precise, and region-specific data are imperative for ensuring accurate outcomes. Furthermore, patients' apprehensions regarding data confidentiality pose hurdles in compiling data effectively. While integrating AI with RAS introduces decision-making paradigms, achieving complete automation remains a subject of ongoing research, hampered by issues such as misclassification and computational constraints [43].

#### 3.1.3 Tactile internet (TI) and internet of things (IoT)

TI and IoT-related studies have become an indispensable aspect of robotic surgeries. As previously highlighted, datasets concerning robotic surgeries encounter significant challenges, including data acquisition and storage complexities, communication difficulties with low-latency requirements, and the scarcity of annotated data [44]. Furthermore, there are limitations in converting structured data into digitized formats. Therefore, it is paramount to consider these studies at the tactical level of OM-RAS.

Gupta et al. [24] introduced the Blockchain-driven Intelligent scheme for Tele-surgery System (BITS) framework, leveraging 6 G networks and AI to mitigate cyberattacks in RATS. BITS addressed limitations in earlier systems like blockchain-based secure and flawless interoperable tele-surgery (HaBiTs), which relied on 5 G and private blockchain but faced issues with latency and reliability [45]. BITS achieved superior performance—99.99 % reliability and sub-100 microsecond latency—enhancing data throughput and reducing storage costs. These advancements improved the responsiveness of intra-operative imaging and haptic feedback tools (e.g., Omega series), enabling more precise real-time surgical control.

To further improve network longevity and security, P. Lokhande & D. Patil [36] proposed a Machine-to-Machine (M2M) communication system using a LEACH (protocol that does not consider cyber-attacks). By simulating cyberattacks through the LEACH-A protocol, they evaluated metrics like energy consumption, PDS, delay, and overhead, demonstrating a 20–25 % increase in service life. Such enhancements support the seamless integration of advanced technologies into telesurgical workflows, ensuring secure, efficient, and real-time data exchange critical to surgical precision.

Hentati et al. [35] explored resource allocation in tele-surgery over 5 G networks by implementing a Joint Placement and Scheduling Algorithm (JPSA) for Virtual Network Functions (VNFs) within a Network Function Virtualization (NFV) environment. Using a greedy algorithm and Integer Linear Programming (ILP), the study prioritized reliability and latency. Without existing benchmarks, two reference algorithms, RM-FDFS and SFG-JPSA, were developed. JPSA outperformed both in admission rate and cost, especially under strict reliability constraints. Notably, splitting application traffic into multiple VNF forwarding graphs (VNF-FGs) with varying QoS demands proved more efficient than using a single VNF-FG.

This insight aligns with Aripin et al. [23], who introduced the "Hospital of the Future" (HoF) concept by implementing three differentiated 5 G network slices tailored to specific communication needs detected via AR. Comparing static and dynamic slicing strategies using micro-BS/femtocell setups, they evaluated performance based on client connection metrics and bandwidth utilization. Although costlier, dynamic slicing with femtocell yielded better outcomes, supporting advanced TS, AR-assisted robotic monitoring, and remote care, key to realizing the HoF vision.

#### 3.1.4 Clinical utility and resource allocation

In a clinical utility context, Mukherjee & Sinha [26] examined RAS scheduling to minimize costs by analyzing three operational policies: triaging patients by criticality, optimizing surgeon pool size, and considering surgeon experience. Using Generalized Linear Models (GLM), Mixed Integer Linear Programming (MILP), and Discrete-Event Simulation (DES), the study found that applying these policies—particularly for complex cases like uterine sizes >150 *g*—improved outcomes and reduced costs in Robot-Assisted Hysterectomy (RAH). However, the authors noted these policies may vary across procedures, such as Radical Prostatectomy (RP), where additional factors like urinary incontinence, blood loss, erectile dysfunction, and urethral complications must be considered. More about results and assumptions can be seen in Table 5.

**Table 5:** Surgical robotics operations and workflow optimization.

Study	Level	Focus		Objective		Methodology	Main Outcome
Lipsitz et al. [32]	Strategic	RAS Integration in surgical practice		Operational comparison (LOS): RAS vs. open vs. lap		Comparative analysis using WLS and log-log link	Indicated shorter LOS of RAS
Al-Thani et al. [28]	strategic	RAS Integration in surgical practice		Operational comparison (LOS, and post-operative complications): RAS vs. open vs. lap		Comparative analysis using retrospective observational study	Indicated shorter LOS of RAS. And open surgery was preferred for larger tumors
Duran et al. [29]	strategic	RAS Integration in surgical practice		Operational comparison (LOS, BL, and morbidity): RAS vs. open vs. lap		Comparative analysis using retrospective observational study	Indicated shorter LOS, less morbidity level, and acceptable BL units of RAS.
Kim et al. [31]	strategic	RAS Integration in surgical practice		Operational comparison (LOS, BL, and morbidity): RAS vs. lap		Comparative analysis using prospective, multicenter comparative study	Indicated equal recovery time, LOS, BL units, mortality level for both RAS and laparoscopic surgeries. However, more OT detected for RAS
Yu et al. [33]	strategic	RAS Integration in surgical practice		Operational comparison (LOS, BL, and OT): RAS vs. lap		Comparative analysis using Clavien-Dindo classification	Indicated equal OT, LOS, BL units, and complications for both RAS and laparoscopic surgeries. However, more medical cost incurred by RAS
Agarwal et al. [34]	Strategic	AI Integration in RAS		Examination of the importance of AI, RAS, and surgeon experience		Benchmark of selected Indian hospitals	Indicated lower treatment cost of RAS with AI
Faria et al. [30]	Strategic	RAS Integration in surgical practice		Clinical and economic comparison (QALYs, ICER, and ICUR): RAS vs. open vs. lap		Comparative analysis using Markov state transition model	Indicated cost-effectiveness of RAS
Ali Mohamad et al. [22]	Strategic	AI Integration in RAS		Examination of the importance of AI in RAS		Dubai case study with semi-structured interviews, archival, and online data analysis	Indicated positive clinical utility outcomes of RAS with AI
Gupta et al. [24]	Tactical	RATS		Communication enhancement in RATS		Blockchain-based tele-surgery framework	Improved network performance with low latency and high throughput
P. Lokhande & D. Patil [36]	Tactical	RATS		Communication enhancement in RATS		M2M communication system through the internet	Improved network performance with reduced energy consumption
Hentati et al. [35]	Tactical	RATS		RATS network resource allocation		ILP, and greedy algorithm	Improved cost-effectiveness
Aripin et (al.2023)	Tactical	RATS		RATS network resource allocation		Simulation	Improved cost-effectiveness
Keyhanian et al. [37]	Operational	Management of RAS resources		Optimization of surgical instruments allocation		Multi-objectives binary IP model	Improved cost-effectiveness through tray minimization
Mukherjee & Sinha [26]	Operational		Management of RAS resources		Optimization of RAS scheduling	GLMs, MIP, and DES models	Improved clinical outcomes through surgeries assignment

AI: Artificial Intelligence; BL: Blood Loss; BS: Base Station; CH: Cluster Head; DES: Discrete Event Simulation; DoS: Denial of Service (A type of cyber-attack, e.g., Man-in-the-Middle attacks); GLMs: Generalized Linear Models; ICERs: Incremental Cost-Effectiveness Ratios; ILP: Integer Linear Program; LEACH-A: Proposed protocol with cyber-attack; LOS: Length of Stay; M2M: Machine-To-Machine Communication; MIP: Mixed Integer Programming; OT: Operative Time; PDR: Packet Delivery Ratio; QALYs: Quality-Adjusted Life-Years; RA: Robot-Assisted; RAS: Robot Assisted Surgery; RATS: Robot-Assisted Tele-Surgery; TI: Tactile Internet; TS: Tele-Surgery; WLS: Weighted Least Squares.

**Table 6:** Surgical robotics performance enhancement.

Study	Level*	Aim of Study	Application	Method/Algorithm	DOF of The Proposed Design	Time/Accuracy/Related Observations
Deilamsalehy & Havens [46]	Inter-operative workspace	Optimizing the orientation and positioning of a surgical robot	A real-world experiment simulation	Holistic approach using AKF algorithm	6DOF	- AKF algorithm has better estimation accuracy and less mean error compared to the standard EKF algorithm
Kabanov et al. [47]	Intra/Inter-operating workspace	Optimizing the orientation and positioning of a surgical robot	Transurethral operation	Denavit-Hartenberg approach and Cauchy-Bunyakovsky-Schwarz inequality	3DOF	Only the degree of deviation from the pivot point has been displayed using graphs
J. Chen et al. [48]	Intra-operative workspace	Optimizing path generation to automate surgical tasks	Peg transfer (for straight paths) and pattern cutting (for curved paths) tasks	RL and LfD	-	The final path trajectory has been detected using GPR by extracting final features from RL and LfD, thus increasing accuracy
Shen [49]	Intra-operative workspace	Optimizing the path of the end-effector	Knee surgery	Credit Assigned Cerebellar Model control and non-dominated genetic algorithm sorting	6-joint robotic manipulator	Proposed design improves the precision of cutting and drilling in knee surgery, optimizes the path of the end-effector, and improves the quality and efficiency of knee surgery
Vairavasamy et al. [50]	Inter-operative workspace	Mechanism optimization and Kinematic analysis	Tele-surgery	VR-simulation	5 DOF Manipulator	Real-time model synchronized and achieved
Trejo & Hu [51]	Intra-operative workspace	Mechanism optimization and Kinematic analysis	Brain tissue dissection-neurosurgery	VR-simulation	neuroArm, with 6DOF	Virtual reality mapping (open surgery) demonstrated superior accuracy and speed. Hypothesis results varied, and the analytic model provided real-time force feedback within 2.5 s
Karadimos et al. [52]	Intra-operative workspace	Mechanism optimization and Kinematic analysis	3 trajectories examined: elbow-up, insertion, and line segment pivot trajectory	VR-simulation and Holistic modeling approach using RRTConnect algorithm	Manipulator has 7DOF	High precision achieved: pivoting accuracy 2.11 µm, repeatability 1.61 µm; insertion accuracy 0.29 µm, repeatability 0.29 µm.
Yang et al. [53]	Inter-operating workspace	Mechanism optimization and Kinematic analysis	Endoscopic sinus surgery	VR-simulation and genetic algorithm to optimize the rod length of the proposed design	3DOF	Omega.7 device provides better manipulation dexterity and accuracy
Yongfeng et al. [54]	Intra/Inter-operative workspace optimization	Mechanism optimization and Kinematic analysis	Lumbar spinal surgery	Genetic algorithm used to optimize the dynamic feature of the proposed robotic design	The Proposed Bi-planar parallel mechanism has 6DOF	macro-micro mechanism provides better accuracy in pedicle screw placement
Du et al. [55]	Inter-operative workspace optimization	Mechanism optimization and Kinematic analysis	Liver tumor	Quantitative: ultrasonic imaging guided medical robot (radio frequency ablation)	The robot arm has 3DOF	Minimum and maximum values of the 3DOF parameter are located to optimize the surgical workspace
Laribi et al. [56]	Intra/Inter-operative workspace evaluation	Mechanism optimization and Kinematic analysis	Anastomosis technique	Inverse and forward kinematics model	–	A cone with a half apex angle (α) and an axis of revolution zT defines the usable workspace
Lu et al. [57]	Intra-operative workspace	Enhancing the efficiency and quality of knot tying through RAS	KT via the experimental tissue pad	Simulating trajectory profile through MATLAB	3DOF manipulator	The model displayed less KT time
Sannikov [58]	Intra-operative workspace	Integrating a laser scalpel into surgical robots	Laser scalpel	2 algorithms used: 1st algorithm to detect the depth map (PID controller used), 2nd algorithm to detect the controlling distance coordinates	–	Integration of 3D camera, depth map tech, real-time PID feedback, and laser adjustments ensures accurate, minimally invasive procedures
A. Takacs et al. [59]	Inter-operative workspace	Building a nonlinear soft-tissue model to mimic a liver-type tissue	Tele-surgery	Heuristic modeling approach and 3 Wiechert model implementations: two non-linear approaches and one linear approach	–	The nonlinear Wiechert model ensures realistic force response, a gradual rise in force, and an accurate representation of stiffness changes

RAS: Robot Assisted Surgery; AKF: Adaptive Kalman Filter; EKF: Extended Kalman Filter; DOF: Degree of Freedom; ML: Machine Learning; RL: Reinforcement Learning; LfD: Learning from Demonstration; GPR: Gaussian Process Regression; SD: Standard Deviation; KT: Knot-Tying; PID: Proportional Integral Derivative; WL: Work Load; DR: Damage Reduction.

⁎ Intra-operative workspace is within a patient's body, while Inter-operative is within an operating room workspace.

Although not focused solely on RAS, Keyhanian et al. [37] proposed a multi-objective binary model for surgical instrument allocation in operating room trays. The model combined cell formation (grouping instruments by use) and bin packing (tray optimization), addressing constraints like avoiding redundancy and managing instruments as limited resources. The authors suggested their approach is adaptable for RAS, offering valuable methodology for efficient surgical logistics.

### 3.2 Surgical robotics performance enhancement

This subsection explores various facets of optimizing the capabilities and efficiency of surgical robotic systems. Each part in this subsection addresses a distinct aspect of performance enhancement.

Our exploration started with an investigation into how surgical robots are controlled in terms of orientation and position (3.2.1). Subsequently, we focused on the optimization of surgical tools and the manipulator's path (3.2.2). The in-depth investigation of the optimization mechanism and kinematic analysis (3.2.3), including the examination of VR-based simulation studies (3.2.3.1). Precision improvement in the manipulation of robotic instruments takes center stage in our analysis (3.2.4), followed by a thorough exploration of imaging enhancement (3.2.5) and the integration of haptic sensing with force feedback (3.2.6). Collectively, these subsections provide a comprehensive overview of ongoing efforts to enhance the performance and capabilities of surgical robotic systems, ultimately contributing to advancements in surgical outcomes and patient care.

#### 3.2.1 Orientation and position of surgical robot

Deilamsalehy & Havens [46] investigated robot pose estimation in RAS using 2D sensors within a 3D simulation to track 6 DOF. Accuracy was assessed through process and measurement noise covariances and mean errors, employing both Extended and Adaptive Kalman Filters. However, their assumption of constant noise covariance limited the model’s adaptability, suggesting future work should explore variable covariance models.

In contrast, Kabanov et al. [47] focused on optimal positioning of a 3 DOF instrument manipulator inside the patient’s body using a linear algebraic method. Accuracy was measured by deviation from the pivot point, providing a distinct approach to evaluating spatial precision in RAS.

#### 3.2.2 Path optimization of surgical tools/manipulators

Path optimization in intra-operative workspace (within the patient body) is another application of interest studied using simulation. J. Chen et al. [48] used AI/ML techniques such as: Reinforcement Learning (RL) and Learning from Demonstration (LfD) to generate an optimized end-effector path by transferring data to Da Vinci system automatically. RL and LfD are used to define paths for two tasks, then Gaussian Process Regression (GPR) is used to detect the final path, and metrics like completion time, path lengths, and average speed are used to examine the proposed model. The path of a 6DOF end-effector in an intra-operative workspace has been optimized by Shen [49] during a knee surgery. The multi-body dynamics approach is employed to examine the transmission properties of the surgical manipulator. The proposed end-effector manipulator displayed better path accuracy and increased efficiency of knee surgery.

#### 3.2.3 Mechanism optimization and kinematic analysis

Mechanism optimization and kinematic analysis, a field with the most studies in this section, reflects the importance of providing the most proper design for surgical robot arms, which indeed affects the surgeons’ performance (better ergonomics) and better surgical outcomes. Du et al. [55], Laribi et al. [56], and Yongfeng et al. [54] did not rely on VR for kinematic analysis. All of them aim to optimize the mechanical structure of each proposed robot/manipulator, and this is to optimize both intra-/inter-operative workspace, which makes it suitable for the patient’s/surgeon’s working environment.

Yongfeng et al. [54] proposed 5R mechanism with 6DOF for a lumbar spinal surgery. Genetic algorithm is used to find the optimal dynamic feature. Similarly, Du et al. [55] proposed radio frequency ablation medical robot with 3DOF arm to optimize the workspace for liver tumor surgery. The author hypothesized that the proposed design can help achieve better treatment. What makes this study special is the reliability to provide surgeons with real-time precise positioning, by ultrasonic imaging to guide the robot.

Laribi et al. [56] crafted a groundbreaking teleoperated system, comprising both a slave and master unit. They effectively used Nexus software to accurately capture motion data. The study defined the robot's usable workspace within a cone, marked by a half-apex angle (α) and an axis of revolution (zT). Notably, the research examined end-effector positioning, optimizing this critical aspect of the robot's functionality. Although this study also explored end-effector path optimization, Laribi et al. [56] mainly focused on developing an advanced kinematic design for their surgical robot.

However, a common thread that tied this study with others in the field was the absence of results concerning accuracy. In essence, what these studies lack is rigorous statistical analyses applied to evaluate the precision and reliability of each designed mechanism. Incorporating such analyses would not only reinforce the validity of their findings but also elevate the overall quality and impact of the research.

##### 3.2.3.1 VR-based simulation studies

Four studies integrated VR into surgical simulations. Vairavasamy et al. [50] developed a VR-based prototype using a 5DOF manipulator for real-time simulation, though surgical validation remains necessary due to missing reliability data. Addressing the Remote Center of Motion (RCM) constraint, Karadimos et al. [52] ensured sub-1 mm Remote Center of Motion (RCM) error to minimize patient force during incisions, analyzing accuracy and repeatability via the RRTConnect algorithm across 10 trajectory trials.

Trejo & Hu [51] explored VR’s role in skill transfer from open surgery to the neuroArm, a 6DOF robotic end-effector. Using both NASA-TLX and objective metrics, tracing accuracy, motion quality (MQ), and damage reduction, they found limited support for their hypothesis, though MQ showed promise.

Yang et al. [53] proposed a sinus surgery robot with a 3DOF double parallelogram mechanism. Using Omega7 for haptic interaction, they optimized rod lengths to enhance virtual performance, advancing VR-based control in robotic procedures.

#### 3.2.4 Precision improvement in robotic instrument manipulation

Several studies, including those conducted by Lu et al. [57], Sannikov [58], and A. Takacs et al. [59] have explored various aspects of robotic instrument manipulation. While these studies lack a unified classification, they collectively contribute to improving the precision of robotic surgical procedures. This paper emphasizes their significance, highlighting their positive impact on achieving enhanced perioperative outcomes in surgery.

Recent advancements in RAS have seen innovative approaches to enhance efficiency and precision. Lu et al. [57] introduced a MATLAB-based method for knot-tying tasks, significantly improving suturing performance and reducing task time. Sannikov [58] integrated a laser scalpel into a surgical robot, enabling real-time corrections based on video feedback, ensuring minimal invasiveness. A. Takacs et al. [59] explored mechanical models for optimal force feedback, emphasizing their crucial role in enhancing precision and safety in RAS.

#### 3.2.5 Imaging

In Table 7, we classify imaging-related studies based on optimization and simulation for inter- and intra-operative applications, identifying 10 studies [[60], [61], [62], [63], [64], [65], [66], [67], [68], [69]] that significantly enhances the performance of RAS. Notably, most of these simulation studies leverage AI techniques, particularly Deep Learning (DL) models, for guidance, classification, or task automation.

**Table 7:** Studies on imaging.

Study	Phase	Sample/Input dataset	Application	Method/Algorithms	Time and Accuracy Results
Shvets et al. [68]	Intra-operative	8 × 75-frame sequences and two full 300-frame sequences	Segmentation of robotic tools	U-net, TernausNet, and LinkNet algorithms	TernausNet-16 excelled in binary segmentation (83.6 % IoU, 90.1 % Dice) and instrument segmentation (65.5 % IoU, 75.9 % Dice). TernausNet-11 led multi-class segmentation (34.6 % IoU, 45.9 % Dice), while LinkNet-34 was the fastest, thanks to its superior encoder
Feng et al. [62]	Pre-operative	- 56 passive sphere places in CT device to obtain CT images - CT images with voxel size 512 × 512 × 1900 - Model trained for 150 epochs	Segmentation of robotic tools (passive marker sphere)	KiU-Net, kite-net, and U-net algorithms	KiU-Net achieved 95.2 % dice accuracy, with significantly fewer parameters than U-net, leading to faster training and lower memory usage
Sivarasa & Jerew [69]	Pre-operative	- 500 × 500 image and 20 × 20 border filters - 16 epochs used to execute the system	Segmentation of robotic tools (forceps parts)	7-layers-CNN	ReLu installation helped increase accuracy by 2 % and decrease processing time by 2s
Nahushev [66]	Intra-operative	Video sequence with 30–60fps frequencies	Segmentation and Localization of tissue abnormalities (e.g. rupture of tissues and blood loss)	To fulfill the application objective proposed algorithm is adjusted to: - Consider that only robotic tools are dynamic. - Detect any motion except robotic tools motion. - Exclude any zones occupied by surgical instruments	Proposed algorithm model enhanced RAS in terms of: Results repeatability and work duration
Glashev [63]	Pre-operative	Previously marked pelvic organs images	Segmentation and Localization of tissue abnormalities (e.g., Sactosalpinx)	Modified-CNN	More training data is needed to increase the accuracy of segmentation
Alqaoud et al. [60]	Pre/intra-operative	- Data obtained from 10 patients - Two MR modalities - Image size: 512 × 512 - First nnU-Net network segmentation used as input for the second network	Segmentation and localization of breast tissues (fat, FGT, and tumor masses)	nnU-Net algorithm with multimodal input	The architecture reduces personnel need, achieving high accuracy (DSC: 0.95±0.00 for breast, fat; 0.83±0.04 for FGT; 0.41±0.58 for tumors)
Padhan et al. [67]	Intra-operative	540 (rtMRI) collected every 50ms	Path optimization and dynamic guidance of robotic manipulators	predefined kinematics and guidance curve, and maneuvering command parameter	Hypothetical clinical task performed using DGVF showed: Better safety (it kept the manipulator within the safety region, 5 mm) and higher accuracy. It also decreased task time by almost 14.8s
Mach et al. [65]	Intra-operative	62 OCT-images of needle-tip annotated volume 5o from different OCT machines and 23 from the local OCT machine	Path optimization of robotic manipulator SNI	3DU-Net algorithm for the segmentation of needle-tip and Levenberg−Marquardt (ML) algorithm	Micron error limit is 25 µm; mean errors in retinal layers, target board, and pig eye evaluations are 23.8 µm, 25.4 µm, and 24.3 µm, respectively, with standard deviations of 5.9 µm and 6.7µm
Dong et al. [61]	Pre-operative	Dataset collected using commercial device $\text{polaris}\;\text{veg}a^{@}$	Path optimization of robotic manipulator and increasing scene adaptivity during RAS	- Smooth Motion Path Planning algorithm: This strategy is useful in resolving the problem of the singular point	Insertion accuracy has an error of less than 1.5mms
Huynhnguyen & Buy [64]	Pre-operative	- From JIGSAWS dataset (39 videos of suturing task conducted by 8 surgeons; each conducted the task 5 times) - frame size 240 × 320 pixels with 10Hz - 10 gestures have been classified	Implementing a suturing task	3-layer 3D CNN to detect the transition between surgeon and LSTM algorithm to classify each gesture	- LOSO for: Gesture transition: around 70 % accuracy. - SGD for: Gesture classification: around 76.3 % accuracy

CNN: Convolutional Neural Network; CT: Computed Tomography; Dice Similarity Coefficient (DSC): A kind of metric used for analysis; DGVF: Dynamic Guidance Virtual Fixtures; IoU: Intersection over Union; JIGSAWS: JHU-ISI Gesture and Skill Assessment Working Set; LOSO: Leave-One-Supertrial-Out (analysis technique); LSTM: Long-Short-Term Memory (analysis technique); MR: Magnetic Resonance; OCT: Optical Coherence Tomography; RAS: Robot Assisted Surgery; ReLu: Rectified Linear Unit; RMSE: Root Mean Square Error; rtMRI: Real-time Magnetic Resonance Imaging; SGD: Stochastic Gradient Descent; SNI: Subretinal Needle Injections; FGT: Fibro Glandular Tissue (A type of breast tissues).

**Table 8:** Studies on haptic sensing and force feedback.

Study	Application/Experiments	Methodology	Manipulator	Observations (E.G., Time, Accuracy Results)
Chioson, Espiritu, Munsayac, Dajay, Jimenez, et al. [70]	Current and PID experimentation has been done to test the performance of the proposed haptic controller	Encoder filtering method	3D printed Single-DOF haptic controller handle	Integral gain aimed to fix steady-state error but caused system instability and slower response; filters and reduced derivative gain further slowed the PI controller
Jiang et al. [71]	Experiments were done on the Spinal Surgery System Robotic (RSSSI) to verify the stability of the proposed model	PSO parameter optimization and Root-Locus method	Experiment conducted on RSSS-II (6DOF serial-link robot)	Novel design optimized control, removing sensors, enhancing human-robot interaction. SMD-System surpassed the proportional controller, ensuring smoother end-effector output and better change rate
Chua et al. [72]	Sinusoidal pulses are used to test the proposed Force feedback model	Physical Model Simulation	–	Higher force caused instability, stronger overshoots, and increased peak overshoots; shorter time intervals between waves led to noticeable overshoots, and time delay increased with shorter intervals
Xie et al. [73]	Testing the force feedback response delay of a master-slave robotic system	Sensor noise filtering (Kalman Filtering algorithm)	Franka Emika manipulator, 7DOF	Omega.7 boasts minimal delays: algorithm and communication <1 ms, grasper closure 10 ms, mechanical 30–40 ms. It excels in high-frequency force signals with <100 ms feedback delay and low error rates
Chioson, Espiritu, Munsayac, Dajay, Santos, et al. [70]	To create a bilateral Direct Force Reflection teleoperation system for a laparoscopic grasper	Mechanism sensing and Sensor noise filtering	1DOF pistol-type haptic device with a maximum force of 1N	PI-controller had low accuracy, possibly due to sensor oscillations or filter issues; temperature fluctuations affected sensor readings; accuracy percentages were 81.42 % (1 N), 75.71 % (2 N), and 91.43 % (3 N)
Safavi & Zadeh [3]	4 subjects of peg transfer were used to validate the model	HMM and LfD	Model-based approach based on a 5DOF Laparoscopic device	Medium MPC excelled in TCT, EoM, and MSM. Vector quantization reduced data size, preserving accuracy. Zero-speed task segmentation improved TCT identification accurately and efficiently
F. Chen et al. [74]	Assessing the function of a cardiovascular interventional master-slave robot during a carotid artery model experiment	Sensor noise filtering (variable limiting filtering)	Master-slave cardiovascular interventional robot	Research focused on z-axis forces for complex aneurysm lesions. Robot-guided wire accurately, showcasing potential for surgery automation, reducing intervention and radiation
Sadeghnejad et al. [75]	Novint Falcon—a parallel impedance-type robot used as a setup for endoscopic sinus surgery	Impedance modeling and MPC, and Quasi-min–max algorithm	Parallel impedance–Novint Falcon robot with 3DOF	A new cost function enhanced model robustness, while the MPC method effectively eliminated disturbances from control signal switches and reduced time delays

EoM: Economy of Motion; F/T: Force-Torque; HMM: Hidden Markov Model; MPC: Model Predictive Control; MSM: Motion Smoothness; P-controller: Proportional controller; PID: Proportional-Integral-Derivative; PI-controller: Proportional Integral controller; PSO: Particle Swarm Optimization; SMD: Spring-Mass-Dashpot System; TCT: Task Completion Time.

**Table 9:** Studies on skill assessment.

Study	Phase	Sample/Input Dataset	Task	Application	Method/Algorithms	Platform	Time and Accuracy Results
El-Saig et al. [76]	Pre-operative	N/A	KT	Assessment through JIGSAWS	Software developed using Peewee Python ORM library	DaVinci platform	The tool can analyze movement paths, identify surgical actions, and process data from JIGSAWS metadata
K. Takacs & Haidegger [77]	Pre-operative	The measured metrics for each of the seven FRS-Dome tasks were used as inputs in the fuzzy systems. (Focus on Psychomotor skills-3rd module)	Two FRS dome tasks: ST, RT	Assessment through FRS dome	ANFIS)	DaVinci platform	Performance of 6 tasks was observed using FRS dome 22 metric, and optimizing results with better accuracy was achieved by developing Neuro-fuzzy Inference Systems for each task
K. Takacs et al. [78]	Pre-operative	37 conducted measurements for only two tasks RT and KT (focus on Psychomotor skills-3rd module)	KT and RT	Assessment through FRS dome	Sensorized FRS Dome. Mounted force-gauge and *C*++ to connect the main program with all connected sensors	DaVinci platform	Tower movement metric for both tasks KT and RT: improved. And tower contact time for RT: improved
Lajko et al. [79]	Intra-operative	JIGSAWS used to obtain kinematic and 2D-visual input data	ST, NP, and KT	Assessment through JIGSAWS	CNN, LSTM, CNN and LSTM (combined), ResNET, and convAuto algorithms	DaVinci platform	The study utilized LOSO cross-validation to prevent overfitting. CNN achieved 80.72 % (ST), 79.66 % (NP), and 80.41 % (KT) accuracy. CNN+LSTM reached 81.58 % (ST), 83.19 % (NP), and 82.82 % (KT), while ResNet scored 81.89 % (ST), 84.23 % (NP), and 83.54 % (KT)

ANFIS: Adaptive Neuro-Fuzzy Inference System.; KT: Knot-Tying; RT: Ring Transfer; ST: Suturing; SVM: Support Vector Machine.

For instance, Shvets et al. [68] employed DL algorithms such as U-net, LinkNet, and ResNet to segment robotic manipulators, improving the surgeon's ability to differentiate between tissue and end-effectors. Feng et al. [62] utilized Kiu-Net to automate 3D segmentation of passive marker spheres. From Shvets et al. [68], U-net might display less Intersection over Union (IoU) or Dice accuracy, however Feng et al. [62] showed its importance in acquiring high-level features, while Kiu-Net was used to capture the finest details.

Sivarasa & Jerew [69] improved tool detection and feature extraction in laparoscopic surgery using a DL-based approach, enhancing accuracy. The proposed solution utilized a 2D convolutional operation similar to the state-of-the-art method by Mikada et al. [80]. Furthermore, a ReLU (Rectified Linear Unit) layer was incorporated, enhancing the system's performance. This modification resulted in a dropout rate of only 20 %, surpassing the state-of-the-art approach by 2 % in terms of accuracy.

In another context, some technology features such as Dynamic Guidance Virtual Fixtures (DGVF) assist surgeons in obtaining intra-operative real-time Magnetic Resonance Images (rtMRI). Such Virtual Features (VFs) used to be conducted based on a pre-operative procedure registering to imitate a real-time UltraSound (US) scene, which might not be suitable for unpredictable movement of tissues. Consequently, Padhan et al. [67] developed on-the-fly DGVF to guide bendable manipulators using Magnetic Resonance Imaging (MRI). Dong et al. [61] optimized puncture paths to enhance scene adaptivity without increasing robot autonomy.

Mach et al. [65] used DL 3DUnet models with Swept-Source Optical Coherence Tomography (SS-OCT) during Subretinal injection surgery, achieving promising results in position identification. The novelty implemented with SS-OCT displayed many advantages over the conventional Time-Domain OCT (TD-OCT).

Huynhnguyen & Buy [64] utilized CNN and Long-Short-Term-Memory (LSTM) models to automate suturing tasks, showing potential for pre-operative automation. Similarly, Glashev [63] employed CNN to autonomously identify diseases associated with pelvic organs. The study introduced image labeling for pathological areas, emphasizing that as the number of labeled images increases, the recognition efficiency also improves.

The nnU-Net algorithms, previously established as state-of-the-art in biomedical tissue segmentation architecture [81], were employed by Alqaoud et al. [60] to propose a groundbreaking approach. They introduced two consecutive nnU-Net networks designed to automatically segment distinct breast tissues, including the breast region, fat, Fibro-Glandular Tissue (FGT), and tumors. Remarkably, their AI model's effectiveness aligns with findings in resource allocation studies (e.g., [34]), indicating that AI implementation can significantly reduce the need for personnel per task due to its autonomous capabilities.

Integrating imaging technology into RAS is crucial, going beyond tool segmentation. In a study by Nahushev [66], a new approach was proposed, instead of the common practice where surgeons manage all tools manually, Nahushev suggests involving an assisting surgeon to maintain a secondary optical channel. This innovative method aims to decrease OT by parallelizing tasks. The core concept revolves around real-time detection of tissue abnormalities during the surgery, using imaging to identify any motion unrelated to tools or physiological fluctuations. This approach represents a significant advancement in RAS, enhancing precision and efficiency.

Integrating imaging technology into RAS plays a vital role in improving various aspects, such as tool segmentation, scene adaptivity, and task automation, ultimately reducing surgical risks and complications.

#### 3.2.6 Haptic sensing and force feedback

Promising advancements such as enhanced ergonomics, reduced surgeon fatigue, improved tremor control, and immersive 3D visualization have become apparent through RAS. However, a notable drawback persists: the absence of haptic sensation and force feedback during RAS. This critical gap is highlighted as one of the factors affecting OM-RAS, emphasizing its significance in the scope of RAS.

In response to this challenge Chioson, Espiritu, Munsayac, Dajay, Jimenez, et al. [70] ingeniously developed a 1-DOF tactile controller providing 1-N force feedback for palpation. Their study revealed that employing numerous filters slowed down the PI controller responses. Conversely, Jiang et al. [71] proposed an innovative Mass-Spring Dashpot (SMD) model, replicating cat muscle and capable of sensing any force acting on the robot. Parameters for this model were optimized using the Particle Swarm Optimization (PSO) algorithm, showcasing superior performance over the P-controller when integrated into the RSSS-II (6DOF serial-link robot).

Chua et al. [72] used MATLAB to simulate a mass-spring force feedback model in a master-slave robotic system, applying sinusoidal pulses between 2 N and 5 N. Their study emphasized the lack of real-life datasets, particularly for systems like DaVinci, limiting simulation accuracy. Similarly addressing force feedback challenges, Xie et al. [73] employed Omega.7 to capture 3D hand motions and forces up to 8 N. Using Kalman Filtering, they reduced feedback delay to under 100 ms while maintaining relative error below 3 % and absolute error under 0.1 N by accounting for mechanical, communication, and algorithmic delays.

In related work, Chioson, Espiritu, Munsayac, Dajay, Santos, et al. [82] integrated force sensors into a laparoscopic grasper for a teleoperation system with bilateral control, testing haptic feedback at 1 N, 2 N, and 3 N. Despite strain gauge thermal limitations, the 3 N force yielded the highest accuracy, with filtering affecting PI-controller precision but ambient temperature having no significant effect.

Further advancing force feedback models, F. Chen et al. [74] developed a carotid artery model with three aneurysms, incorporating a 6DOF F/T sensor and real-time 3D imaging. The study found maximum resistance along the z-axis, with minimal resistance in the x and y directions.

Building on recent advances, Safavi & Zadeh [3] introduced a model-based force rendering approach (MPC—HG) using a 5DOF laparoscopic device with force sensors and a Bakis-type Left-to-Right Hidden Markov Model (HMM) trained via Learning from Demonstration (LfD). This method effectively captured the non-deterministic nature of surgical motions. Among four evaluated control modes, their Model Predictive Control (MPC), modeled through a Multi-Layer Perceptron (MLP) network, achieved superior results in Task Completion Time (TCT) and Economy of Motion (EoM), marking a breakthrough in handling system complexities and uncertainties, as evidenced by the work of Golnary & Moradi [83] and Vrooijink et al. [84].

Extending this line of work, Sadeghnejad et al [75] developed a 1DOF mass-stiffness-damping model with a five-parameter impedance framework to simulate the human arm in a VR-based Endoscopic Sinus Surgery trainer. Using a quasi-min–max output feedback MPC for improved robustness, they addressed uncertainty and worst-case dynamics. A phenomenological tissue fracture model and the 3DOF Novint Falcon manipulator enhanced realism and control. Following noise reduction and delay compensation, the simulator delivered strong performance, reinforcing its value for surgical training.

Traditionally, surgeons utilize palpation to assess tissue characteristics, locate nerves and arteries [85], and identify irregularities like lumps [86,87]. In addition, they depend on their sense of touch to control the amount of pressure applied. Applying excessive force can result in tissue damage, internal bleeding, and broken sutures. Conversely, insufficient force can lead to loosely tied knots and inadequate sutures [88,89]. However, advancements mentioned previously in this section underscore the relentless pursuit of refining haptic sensing and force feedback technologies that impact parameters such as suturing time, which affect the overall OT of the surgery.

### 3.3 Skill assessment

Surgeons with over twenty robotic procedures demonstrated superior perioperative outcomes and fewer complications compared to in-training surgeons in their initial twenty robotic surgeries [90]. Unfortunately, there are no standardized programs for RAS training [91], which reflects the importance of addressing skill assessment in a separate section.

Skill assessment in the context of RAS has been studied more frequently through the perspectives of AI in robotic surgeries [15], ML of technical skill assessment [8], or real-time skill assessment of robotic surgeries [92]. However, no study has examined how AI, ML, or real-time techniques are going to affect OM of RAS, which explains the scarcity of studies in this section. Using the search query described in the methodology section, only four studies were found [76,79,78,77].

Growing evidence suggests that the technical abilities of surgeons impact the outcomes of patients after surgery, as supported by numerous studies [[93], [94], [95]]. Implementing techniques to assess these skills and offer feedback during surgeons' learning processes can enhance training efficiency [96,93]. Therefore, assessment through JAW or dome is introduced.

JIGSAWS (JHU–ISI Gesture and Skill Assessment Working Set) is an open-source annotated dataset of eight surgeons from three degrees of expertise doing 103 basic robotic tasks related to RAS, which are: suturing, knot-tying, and needle-passing. Such a database is very beneficial in skill assessment; on the other hand, processing data from JIGSAWS might be complicated due to the huge data stored (e.g., kinematic data, video data), which requires proposed approaches of how to analyze such data or use it to assess/classify novice surgeons.

Reaching to a point to analyze such a huge dataset and then classify surgeons will provide severe benefits such as: decreasing the senior surgeons required to assess trainees, and this has a direct impact on OM-RAS in terms of decreasing cost and the number of personnel required to supervise training sessions, in addition it will provide objective assessment rather than subjective. Along with the JIGSAWS data set, the Fundamentals of Robotic Surgery (FSR) dome is also presented in this section, which is believed to exhibit promising results in improving classification procedures of surgeons.

El-Saig et al. [76] developed a graphical tool using Peewee Python to automate skill assessment by analyzing JIGSAWS data, specifically evaluating velocity during knot-tying tasks across varying expertise levels. The tool, comprising two modules: enter-staej.py and main.py, that classifies surgeons and recognizes surgemes.

Lajko et al. [79] expanded this approach by applying five machine learning models to assess surgeon performance without intermediate classes, minimizing misclassification [97]. Their method uniquely incorporated both 2D visual and kinematic data from JIGSAWS.

K. Takacs & Haidegger [77] validated the FRS-dome, a sensorized psychomotor training tool, using two novice-friendly tasks: suturing and ring transfer, then applied an Adaptive Neuro-Fuzzy Inference System (ANFIS) to refine classification boundaries over time using ongoing skill data. In related work, K. Takacs et al. [78] introduced a surgical phantom based on the modified FRS-dome, evaluating technical skill through tower movement and contact time in 37 ring transfer and knot-tying trials.

The DaVinci system remains dominant in OM-RAS, largely due to its rich datasets and integration with tools like JIGSAWS and FRS-dome, enabling robust skill assessment. As highlighted by Mukherjee & Sinha [26], optimizing the surgeon pool is a critical policy in surgery scheduling. Automated assessment tools can reduce personnel demands, improving operational efficiency and directly benefiting OM-RAS outcomes.

## 4 Current challenges and future research paths

**Fig. 4:**
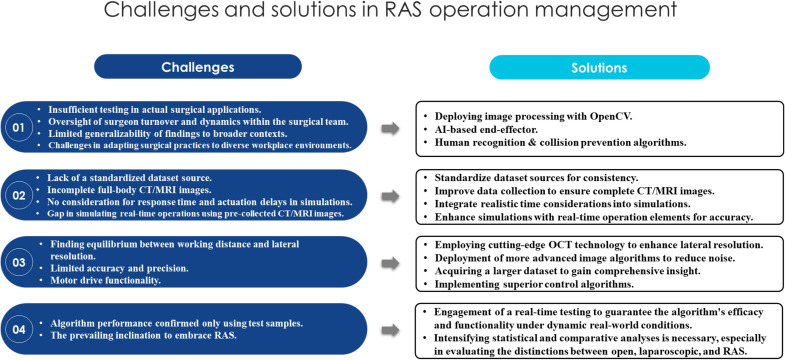
Framework of Gaps and Solution Approaches.

The previous section reviewed efforts to improve OM-RAS. While researchers have built on earlier work to close some research gaps, Table 10 and earlier discussions show that some issues are still unresolved, there are repeated efforts, and some suggested areas haven’t been explored yet. Fig. 4 summarizes the main research gaps and ongoing challenges, along with key requirements for addressing them. This framework is inspired by Hadid, Elomri, Mekkawy, et al. [98], who used a similar method to identify gaps in managing outpatient chemotherapy. Based on this review and the proposed solution framework, this section highlights some challenges and directions for future research.

**Table 10:** Limitations and Future Research Paths.

Study	Limitations	Future Suggestions
Vairavasamy et al. [50]	- The reliability of the proposed model needs to be tested on a real surgical application	- Deploying image processing with OpenCV software - AI-based end-effector - Human Recognition and Collision Prevention algorithms
Keyhanian et al. [37]	- Not mentioned	- The same approach holds significant promise in the context of robotic surgeries
Mukherjee & Sinha [26]	- Analysis did not consider surgeons' turnover and dynamics of the surgical team	- Developing dynamic scheduling algorithms - Predictive analytics models
	- Lacks generalizability	- Considering more constraints to conceive more robotic surgeries, such as radical prostatectomy
Faria et al. [30]	- The use of diverse resources, which affects uncertainty	- The use of standardized protocols and skilled personnel
Feng et al. [62]	- Uniform source of datasets - Incomplete CT images of the complete human body	- Obtaining CT images from different CT apparatuses - Improve data augmentation techniques
Nahushev [66]	- Algorithm performance confirmed using only the test sample	- Conducting real-time testing is necessary to ensure the algorithm's functionality and effectiveness in real-world, dynamic conditions
Padhan et al. [67]	- Real-time operation was simulated using previously collected MR images - Response time and actuation delays are not considered in simulations	- Establishing a real-time connection with an MR scanner to conduct live tests - Considering algorithms to compensate for response time and actuation delays
Mach et al. [65]	- The trade-off between working distance and lateral resolution causes some difficulties in detecting the needle tip and displays some noise	- Deployment of advanced OCT technology that provides better lateral resolution - Deployment of more sophisticated image algorithms that reduce noise
Dong et al. [61]	- Limited workspace adaptivity - Mismatch between Parameters and Reflecting Balls	- Enhancing scene adaptivity by deploying sensors - Deployment of an advanced optical tracking system
[63,64,78,77]	- Limited accuracy	- Larger dataset needed
Chioson, Espiritu, Munsayac, Dajay, Jimenez, et al. [70]	- Lack of precision - Motor-derived limitation	- Requires better control algorithms
Jiang et al. [71]	- Zero drift issues - Neglecting conversion rate analysis	- Implement adaptive control algorithms - Including visual inputs
Chua et al. [72]	- Lacks variable gain values - Real-life input signals are needed to mimic a robotic system	- Need a Hybrid System with Variable Gain - Real-world data integration

### 4.1 Current challenges

#### 4.1.1 Lack of annotated data

Improving robotic surgeries still faces major challenges, especially when it comes to integrating AI, which needs large amounts of metadata. Collecting, storing, processing, and retrieving this data, like from the JIGSAWS database, is slow and difficult, mainly because there's not enough annotated data. Some studies have suggested using 6 G networks to improve data infrastructure [24], but this approach still needs more research.

#### 4.1.2 RAS malfunctions

Malfunctions in robotic surgery can result from software glitches, mechanical failures, or faulty instruments. While Da Vinci system failures are rare, they can still be affected by the surgeon’s experience and how well they respond [99]. To reduce the risk, it’s important to inspect the robotic system carefully before each procedure [100]. More research is also needed to better understand instrument failures. One common cause appears to be wear on the insulating membrane, which can happen due to friction or collisions inside the body or during insertion through trocars [99].

Ethical and legal questions also remain unresolved, particularly around who is responsible when something goes wrong: the manufacturer or the surgeon. Ferrarese et al. [99] described the malfunction reporting process, where hospitals inform the manufacturer, who then notifies the U.S. FDA. However, there is still not enough data on the legal aspects of these cases, highlighting the need for further study into liability issues.

#### 4.1.3 Tendency to adopt new technologies

This review highlights several emerging technologies, including autonomous surgical tasks, AI integration in healthcare, and the growing use of RAS. While many studies show increasing interest in these innovations [34,22,70], their widespread adoption remains uneven. Developing countries face notable challenges in implementing these technologies, and even in Europe, adoption of RAS technologies lags behind the U.S [101]. This makes it essential to examine the barriers to broader implementation from different perspectives.

Another key barrier is the lack of agreement on whether RAS is truly superior to traditional open or laparoscopic surgeries. A bibliometric analysis could help by reviewing the existing literature, identifying key challenges, and highlighting global progress in robotic procedures. This kind of analysis also enables benchmarking and cross-country comparisons, offering a clearer picture of how the field is evolving [102].

### 4.2 Future research paths

#### 4.2.1 RAS automaticity

The field of automated tasks in RAS is still in its infancy and requires further research to establish a solid foundation. More extensive studies are needed to determine the accuracy level that can match human proficiency. The DaVinci system, widely used in robotic surgery, is not entirely autonomous. As a result, researchers have made efforts to automate various aspects of surgical tasks, including repetitive tasks, complex procedures like suturing [64], skill assessment [77], 3D-guidance, and 3D-segmentation [62]. Despite its potential, this area of study is not yet firmly established. Additionally, research has shown that robotic system failures can result from inadequate communication [103]. We also highlight that a restricted intra-operative workspace can lead to complications. Overcoming these obstacles and improving task automation in RAS is essential for increasing the efficiency and effectiveness of robotic surgical operations.

#### 4.2.2 Comprehensive scheduling models

The operational pathway is critical to consider in RAS because RAS procedures are often scheduled for specific surgeries believed to offer superior outcomes compared to traditional methods. However, the setup for RAS requires a significant footprint and entails a prolonged setup time, which can impact the scheduling process significantly. Therefore, optimizing the operational pathway is essential to ensure efficient utilization of resources and minimize delays in scheduling, ultimately enhancing the delivery of robotic surgical care.

This review highlights a gap in how RAS procedures are scheduled. So far, only one study has explored RAS scheduling in depth, showing how it can improve clinical outcomes. Scheduling RAS is complex, which makes it hard to apply a single approach across all surgeries. For example, Mukherjee & Sinha [26] focused on scheduling for hysterectomy and considered specific factors like uterine size. Other procedures, like partial nephrectomy or radical prostatectomy, involve different conditions and require customized scheduling strategies. This shows the need for further research in this area.

Therefore, it is essential to account for factors such as the allocation of operating rooms, the availability of beds, and the optimized number of nurses required for each surgery. Furthermore, integrating clustering into the scheduling process can indeed improve the efficiency of various operations, including surgeries [104].

#### 4.2.3 Simulation models

Along with optimization, simulation models can provide insights into scheduling policies, appointment planning, scheduling, and resource-to-patient assignments, which enhance overall performance. For example, using the Simulation-based Multi-objective Optimization (SMO) approach, which can deal with complexities related to RAS scheduling, capacity planning, and resource allocation [105].

#### 4.2.4 Follow-up period

A postoperative follow-up period can be implemented to assess outcomes and offer necessary care in the event of complications following RAS. Home care not only reduces the frequency of hospital visits but also ensures that essential care is promptly delivered [106].

#### 4.2.5 Tele-surgeries

Although several pioneering studies on using cutting-edge technologies, including 6 G and blockchain, to enhance the tele-surgeries performance, the current research landscape requires more targeted efforts. Specifically, there is an urgent need for additional technical studies, particularly those focusing on data security and the seamless transformation of data. This emphasis on technical details is vital to advancing the field of tele-surgeries effectively. The challenge of telecommunication in telesurgery is significant, as surgical latency inherently increases with greater distances and variability within transmission networks. As the distance grows, transmission efficiency decreases, resulting in longer audio and video latency. This increased latency can hinder the surgeon's ability to synchronize their movements with the actions of the remote robotic system and the patient, potentially disrupting coordination. Robotic telesurgery is increasingly recognized as a transformative approach to surgical care, leveraging advanced telecommunications technologies to enable remote operations. The systematic review by Reddy et al. [107] emphasizes the progression of telesurgery and current capabilities facilitated by high-speed 5 G and fiber-optic networks. Despite technical success, significant barriers persist, including latency challenges, cybersecurity threats, and the absence of universally accepted ethical and regulatory guidelines. These non-technical considerations remain critical obstacles to the broader adoption of telesurgery​​.

Ethical challenges in telesurgery involve patient autonomy, confidentiality, and informed consent, as remote surgeries amplify traditional ethical dilemmas. Technical aspects like data compression and latency variability due to differing telecommunication networks complicate real-time operations. Moreover, concerns around cybersecurity, such as network breaches and unauthorized data access, highlight the importance of robust encryption and multifactor authentication systems. Financial constraints and a lack of consistent reimbursement frameworks further impede telesurgery's scalability, particularly in underserved regions [108]​​.

The future of telesurgery depends on progress in 6 G networks, AI-powered predictions, and augmented reality tools that provide real-time feedback. Dohler et al. [109] highlight the potential of 6 G networks to further minimize latency while integrating AI for enhanced surgical precision and predictive diagnostics. Unified legal frameworks and interdisciplinary collaboration are critical to addressing regulatory and operational gaps. With strategic investments in infrastructure and ethical oversight, telesurgery could redefine global healthcare by bridging disparities and enhancing access to specialized surgical expertise​.

## 5 Conclusion

This comprehensive review investigates the evolving landscape of RAS, shedding light on its multifaceted aspects and the transformative impact of cutting-edge technologies. Rapid advancements in medical innovation have propelled RAS to the forefront of surgical procedures, offering enhanced functional outcomes, reduced operation time, shorter hospital stays, and improved patient recovery. The integration of RAS into healthcare systems, as part of the Healthcare 5.0 paradigm, has guided in promising improvements in peri‑operative outcomes, ranging from reduced waiting times to optimized resource utilization.

However, this transformative journey is not without challenges. The study emphasizes the critical need for a holistic approach, integrating technologies like AI, kinematics, imaging, and the IoT to optimize RAS implementation. It highlights the gaps in current research, urging further exploration into areas such as RAS singularity, scheduling complexities across diverse procedures, and risks associated with nerve-sparing techniques. The literature review underscores the imperative for a multi-criteria decision-making approach, acknowledging that the clinical utility of RAS extends beyond AI-related innovations. It advocates for a meticulous analysis of kinematic intricacies, imaging advancements, and real-time data processing, culminating in a paradigm shift in the research landscape.

Furthermore, the study highlights the pivotal role of OM in steering the success of RAS implementation. OM-RAS is a multidimensional challenge, encompassing complicated aspects such as workflow optimization, performance enhancement, skill assessment, and cost-benefit analyses. While current research predominantly emphasizes technological advancements and simulations, the review spotlights the need for a balanced focus on healthcare logistics, skill evaluation, and cost-effectiveness to realize the full potential of RAS in clinical practice.

The scarcity of studies explicitly focusing on OM-RAS is evident, and the field remains in the early stages of development, leaving several research gaps unexplored. An examination of the limitations within the reviewed publications has identified three key challenges and five future research avenues, each with various potential sub-directions.

As illustrated, RAS is progressively becoming a crucial element in numerous global healthcare systems, emphasizing the necessary to investigate the operational management aspects of RAS. In essence, this literature review not only consolidates the current state of RAS research but also paves the way for future endeavors. It challenges researchers to explore uncharted territories, bridging gaps in knowledge, and exploring deeply into the nuances of RAS implementation. As healthcare systems continue to evolve towards Hospitals of the Future and Healthcare 5.0, this study serves as a guiding beacon, illuminating the path towards optimized, efficient, and patient-centered Robotic-Assisted Surgeries.

## Data access statement

Research data supporting this publication are available to the corresponding author upon reasonable request.

## Funding statement

The research received funding from the Medical Research Center, Hamad Medical Corporation, Doha, Qatar, through grant number MRC-01–22–310 as part of the IRGC-9 cycle.

## CRediT authorship contribution statement

**Abdullah Riad:** Writing – original draft, Visualization, Software, Methodology, Investigation, Formal analysis, Data curation, Conceptualization. **Majed Hadid:** Writing – review & editing, Writing – original draft, Visualization, Validation, Supervision, Software, Project administration, Methodology, Investigation, Funding acquisition, Formal analysis, Conceptualization. **Adel Elomri:** Supervision, Resources, Project administration, Methodology, Investigation, Conceptualization. **Ahmad Al-Ansari:** Validation, Software, Resources, Investigation, Funding acquisition, Conceptualization. **Mohamed Amine Rejeb:** Visualization, Resources, Investigation, Formal analysis, Data curation, Conceptualization. **Marwa Qaraqe:** Writing – review & editing, Visualization, Resources, Formal analysis. **Sarada Parsad Dakua:** Writing – review & editing, Validation, Supervision. **Abdel Rahman Jaber:** Writing – review & editing, Writing – original draft, Validation. **Abdulla Al-Ansari:** Visualization, Validation, Resources, Funding acquisition, Conceptualization. **Omar M. Aboumarzouk:** Writing – review & editing, Visualization, Validation, Funding acquisition, Conceptualization. **Abdelfatteh EL Omri:** Writing – review & editing, Visualization, Validation, Resources, Project administration, Methodology, Funding acquisition, Conceptualization.

## Declaration of competing interest

The authors declare that they have no known competing financial interests or personal relationships that could have appeared to influence the work reported in this paper.

## Appendix Supplementary materials

Image, application 1
