# Supplementary material for: Advancements and challenges in robotic surgery: A holistic examination of operational dynamics and future directions
Source: Surg Pract Sci. 2025 Jul 6;22:100294. doi: 10.1016/j.sipas.2025.100294 (PMC12280407; doi:10.1016/j.sipas.2025.100294)
Supplement: Supplementary file 1 [file mmc1.docx]

Table S1. Categorization of included studies as observational, real-world validated, or mixed-design.

| **Study** | **Type of the Study** | | |
| --- | --- | --- | --- |
|  | **Observational** | **Real-World Validation** | **Mix-design** |
| Lipsitz et al. (2017) | ✓ |  |  |
| Al-Thani et al. (2022) | ✓ |  |  |
| Duran et al. (2014) | ✓ |  |  |
| Kim et al. (2016) |  | ✓ |  |
| Yu et al. (2014) |  | ✓ |  |
| Agarwal et al. (2020) | ✓ |  |  |
| Faria et al. (2022) |  | ✓ |  |
| Ali Mohamad et al. (2023) |  |  | ✓ |
| Gupta et al. (2020) | ✓ |  |  |
| P. Lokhande & D. Patil (2021) | ✓ |  |  |
| Hentati et al. (2022) | ✓ |  |  |
| Aripin et (al.2023) | ✓ |  |  |
| Keyhanian et al. (2018) | ✓ |  |  |
| Mukherjee & Sinha (2020) |  |  | ✓ |
| Deilamsalehy & Havens (2018) |  | ✓ |  |
| Kabanov et al. (2020) | ✓ |  |  |
| J. Chen et al. (2022) |  | ✓ |  |
| Shen (2019) | ✓ |  |  |
| Vairavasamy et al. (2022) | ✓ |  |  |
| Trejo & Hu (2018) | ✓ |  |  |
| Karadimos et al. (2022) | ✓ |  |  |
| Yang et al. (2019) | ✓ |  |  |
| Yongfeng et al. (2015) | ✓ |  |  |
| Du et al. (2007) | ✓ |  |  |
| Laribi et al. (2013) | ✓ |  |  |
| Lu et al. (2016) |  | ✓ |  |
| Sannikov (2019) | ✓ |  |  |
| A. Takacs et al. (2015) |  | ✓ |  |
| Shvets et al. (2018) |  | ✓ |  |
| Feng et al. (2022) |  | ✓ |  |
| Sivarasa & Jerew (2020) |  |  | ✓ |
| Nahushev (2019) |  | ✓ |  |
| Glashev (2019) | ✓ |  |  |
| Alqaoud et al. (2022) |  | ✓ |  |
| Padhan et al. (2022) |  | ✓ |  |
| Mach et al. (2022) |  | ✓ |  |
| Dong et al. (2019) |  | ✓ |  |
| Huynhnguyen & Buy (2021) |  | ✓ |  |
| Chioson, Espiritu, Munsayac, Dajay, Jimenez, et al. (2020) |  | ✓ |  |
| Jiang et al. (2016) |  | ✓ |  |
| Chua et al. (2022) | ✓ |  |  |
| Xie et al. (2020) |  | ✓ |  |
| Chioson, Espiritu, Munsayac, Dajay, Santos, et al. (2020) |  | ✓ |  |
| Safavi & Zadeh (2015) |  | ✓ |  |
| F. Chen et al. (2018) |  | ✓ |  |
| Sadeghnejad et al. (2023) |  |  | ✓ |
| El-Saig et al. (2018) | ✓ |  |  |
| K. Takacs & Haidegger (2021) |  | ✓ |  |
| K. Takacs et al. (2020) |  | ✓ |  |
| Lajko et al. (2021) |  | ✓ |  |
| Total | 22 | 24 | 4 |
